# Editorial note – *Eurosurveillance* articles automatically deposited at PubMed Central

**DOI:** 10.2807/1560-7917.ES.2016.21.40.30364

**Published:** 2016-10-06

**Authors:** Eurosurveillance editorial team

**Affiliations:** 1 European Centre for Disease Prevention and Control (ECDC), Stockholm, Sweden

Since early August 2016, the final version of all *Eurosurveillance* articles are automatically deposited with PubMed Central (PMC). This will facilitate for authors who have hitherto had to make an author deposit in order for their manuscripts to appear in PMC. *Eurosurveillance* is a fully participating journal to PMC, described as ‘a free full-text archive of biomedical and life sciences journal literature at the US. National Institutes of Health's National Library of Medicine’ on their website.

With this, *Eurosurveillance* further supports scientists in complying with public access polices by funders that include peer-reviewed journal manuscript deposition in Open Access archives.

